# Correction: Alanine-scanning mutagenesis library of MreB reveals distinct roles for regulating cell shape and viability

**DOI:** 10.1371/journal.pgen.1012154

**Published:** 2026-05-20

**Authors:** Suman Maharjan, Ryan Sloan, Jada Lusk, Rose Bevienguevarr, Jacob Surber, Randy M. Morgenstein

In Fig 4, the X-axis label in panel C is incorrect. Please see the correct Fig 4 here.

**Fig 4:**
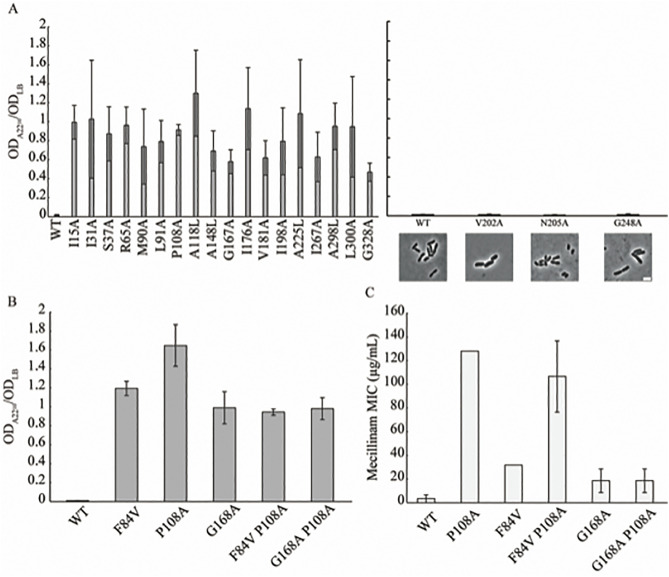
Round mutants do not require elongasome activity for PG synthesis. A-B) Cells were grown for six hours in LB or LB + A22 (10 µg/mL). The ratio of the OD_600_ between the two conditions was determined as a growth ratio. A) A22 growth ratios of the 18 mutants with the most extreme cell shape defects. B) Growth ratios of the rod-shaped suppressors and parental strains. C) Minimum inhibitory concentration to mecillinam of the suppressor mutants and parental strains. All data shown are the average values from three independent experiments with standard deviation‌‌.
